# Distinct regulation of Maf1 for lifespan extension by Protein kinase A and Sch9

**DOI:** 10.18632/aging.100727

**Published:** 2015-02-22

**Authors:** Ying Cai, Yue-Hua Wei

**Affiliations:** ^1^ No. 3 People's Hospital Affiliated to Shanghai Jiao Tong University, School of Medicine, Shanghai 201900, China

**Keywords:** lifespan, yeast, Protein Kinase A (PKA), Sch9, Maf1

## Abstract

The Protein kinase A (PKA) and Sch9 regulates cell growth as well as lifespan in *Saccharomyces cerevisiae*. Maf1 is a RNA polymerase III (Pol III) inhibitor that tailors 5S rRNA and tRNA production in response to various environmental cues. Both PKA and Sch9 have been shown to phosphorylate Maf1 *in vitro* at similar amino acids, suggesting a redundancy in Maf1 regulation. However, here we find that activating PKA by *bcy1* deletion cannot replace Sch9 for Maf1 phosphorylation and cytoplasmic retention; instead, such modulation lowers Maf1 protein levels. Consistently, loss of *MAF1* or constitutive PKA activity reverses the stress resistance and the extended lifespan of *sch9Δ* cells. Overexpression of *MAF1* partially rescues the extended lifespan of *sch9Δ* in *bcy1Δsch9Δ* mutant, suggesting that PKA suppresses *sch9Δ* longevity at least partly through Maf1 abundance. Constitutive PKA activity also reverses the reduced tRNA synthesis and slow growth of *sch9Δ*, which, however, is not attributed to Maf1 protein abundance. Therefore, regulation of lifespan and growth can be decoupled. Together, we reveal that lifespan regulation by PKA and Sch9 are mediated by Maf1 through distinct mechanisms.

## INTRODUCTION

Protein kinase A (PKA) and Sch9 belong to the ACG family of Serine/Threonine kinase [1]. These two kinases are highly conserved throughout evolution [2]. In yeast, PKA consists of three catalytic subunits Tpk1, Tpk2 and Tpk3 and a regulatory subunit Bcy1, while mammals contain two regulatory and two catalytic subunits. The yeast Sch9 kinase is homologous to S6 kinase 1 (S6K1) in mammals [3], a bona fide substrate for mammalian target of rapamycin (mTOR) kinase complex 1, or mTORC1. Consistently, Sch9 is phosphorylated by TORC1 [3], the yeast counterpart of mTORC1. Both kinases are well known to regulate cell growth and proliferation, largely through enhancing ribosome biogenesis [4, 5]. In response to nutrient or growth factors, PKA is activated by cyclic AMP and transduces the growth signals to ribosomal biogenesis machineries e.g. RNA polymerases (Pol Is) including Pol I that is responsible for synthesis of ribosomal RNA (rRNA), Pol II ribosomal protein (RP) and Pol III transfer RNAs (tRNAs). Similarly, Sch9 and its mam-malian homolog S6K1 are also activated by nutrients and growth factors, but through TORC1-dependent phosphorylation [3]. Sch9/S6K1 then targets ribosomal protein S6 and other regulators to modulate protein translation initiation and ribosome biogenesis. Both kinases have been implicated in cancerous transformation [6, 7].

PKA and Sch9 are also well known to modulate lifespan in various organisms [8]. In yeast, reduced PKA activity by deleting any of the catalytic subunits lengthens lifespan [9]. Consistently, modulating upstream activators such as Ras, Cdc25 or Cyr1 also affect lifespan [9–11]. In mice, lifespan and health can be enhanced by deleting a positive regulatory subunit of PKA [12]. Sch9/S6K1 is broadly involved in lifespan regulation: deletion or knockdown of Sch9/S6K1 can extend lifespan in yeast [13, 14], worms [15, 16], flies [17] and mice [18]. However, as part of the mTOR pathway, the detail mechanisms underlying Sch9/S6K1's role in lifespan regulation is unclear [19]. It is intriguing that lifespan are linked to growth by PKA and Sch9, but whether these two biological traits are regulated through the same mechanisms is unknown.

Recently, Maf1 has been identified to be a substrate for both PKA and Sch9 kinases. Maf1 is a key regulator of Pol III-dependent transcription conserved from yeast to mammal [20, 21]. It inhibits the transcription [22, 23] through binding to the target genes including tRNA genes [24, 25]. Maf1 mediates diverse stress signals to regulate Pol III-dependent genes, including DNA damage signals, nutrient signals etc. [26]. Mechanistically, Maf1 has been reported to be controlled through phosphorylation and nucleus-to-cytoplasm translocation. In yeast, Maf1 is localized in the cytoplasm under nutrient-rich conditions [27–29]. In response to starvation or rapamycin treatment, however, Maf1 is dephosphorylated by type 2A protein phosphatases (PP2A) and accumulates in the nucleus. In mammalian cells, Maf1 is only present in the nucleus [30]. Regulation of phosphorylation in the nucleus by TORC1/mTORC1 and PP4 are essential for Maf1 activity [31–33]. At the organismal levels, Maf1 knockdown in *Drosophila* fat body boosts the development and increases the larvae body size [34], demonstrating that Maf1 is a critical to growth control. Both PKA and Sch9 have been reported to regulate Maf1 cytoplasm-to-nucleus translocation. Furthermore, *in vitro* kinase assay demonstrate that both phosphorylate Maf1 at similar amino acids [27, 35–37], leading to a prevailing model that these two kinases function redundantly to regulate Maf1 and Pol III-dependent transcription. However, whether PKA and Sch9 target the same amino acids on Maf1 for phosphorylation *in vivo* remain unclear. If so, whether these two kinases regulate Pol III-dependent transcription through redundant function or through distinct mechanisms is unknown. Furthermore, since PKA and Sch9 are critical to lifespan regulation, whether Maf1 can mediate such biological process is an interesting question yet to be answered.

To address these issues, we have modulated the activity of PKA and Sch9 in yeast cells in order to compare their roles in regulating Maf1 phosphorylation, subcellular localization, protein levels and Pol III-dependent transcription of tRNA. We found that Sch9 but not PKA is majorly responsible for Maf1 phosphorylation and cytoplasmic localization, demonstrating that these two kinases do not function redundantly *in vivo*. Upon further investigation, we found that Maf1 protein amount is cooperatively controlled by both PKA and Sch9 for lifespan regulation and stress resistance, but not Pol III-dependent transcription and growth. We have therefore identified Maf1 as an important modulator of lifespan and revealed distinct mechanisms of regulation by PKA and Sch9.

## RESULTS

### PKA is not redundant to Sch9 in Maf1 phosphorylation and subcellular distribution

**Figure 1:**
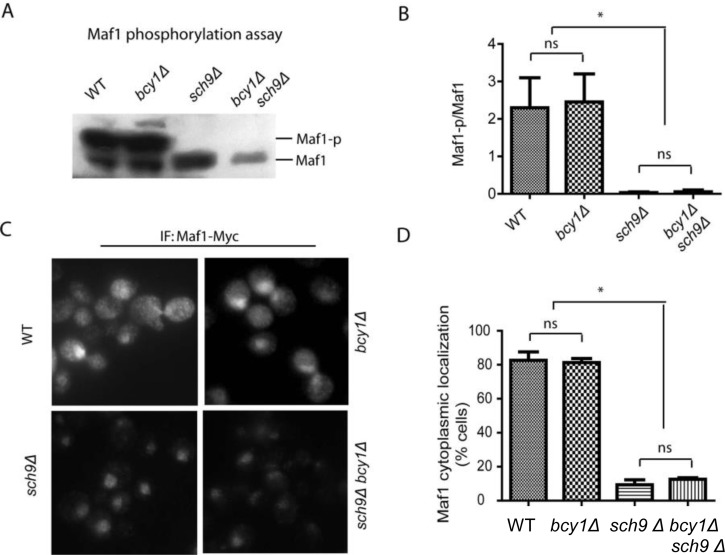
PKA is dispensable for Maf1 phosphorylation and subcellular localization (**A**) PKA hyper-activation does not rescue Maf1 phosphorylation in *sch9Δ* mutant. WT, *bcy1Δ*, *sch9Δ* and *sch9Δ bcy1Δ* cells expressing a Myc-tagged Maf1 (Maf1-Myc) from a low copy centromeric plasmid were cultured to early log phase and protein samples were prepared for western blot analysis. Phosphorylated Maf1 ran slower than dephosphorylated Maf1 on SDS-PAGE. (**B**) Quantification of the ratio Maf1 phosphorylation vs. de-phosphorylation from 3 independent experiments as shown in A. ns, not significant; * *p* < 0.01. (**C**) PKA hyper-activation does not rescue Maf1 cytoplasmic localization in *sch9Δ* mutant. WT, *bcy1Δ*, *sch9Δ* and *sch9Δ bcy1Δ* cells expressing Maf1-Myc from a low copy centromeric plasmid were cultured to early log phase and cells were fixed for immunofluorescence analysis. Maf1 was distributed in both nucleus and cytoplasm in WT and *bcy1Δ* cells but was restricted to nucleus when *SCH9* was further deleted. (**D**) Quantification of cells with Maf1 nuclear localization from 3 independent experiments as shown in C. ns, not significant; * *p* < 0.01.

Both purified PKA and Sch9 can phosphorylate bacterially expressed Maf1 *in vitro* at similar amino acids [27, 35–37]. It has therefore been assumed that PKA and Sch9 may function redundantly *in vivo* to regulate Maf1 and Pol III, hence cell growth. We tested this possibility by asking whether constitutively activating PKA could override the loss of Sch9 for Maf1 phosphorylation. In yeast strain lacking Sch9, Maf1 has reduced phosphorylation [35–37]. If PKA and Sch9 are redundant in Maf1 phosphorylation, constitutive activation of PKA should reverse the reduced phosphorylation of Maf1 in *sch9Δ* cells. Maf1 phosphorylation can be revealed by a retarded species through western blot [28, 29]. Consistent with early studies, loss of Sch9 significantly decreased Maf1 phosphorylation. However, hyper-activation of PKA by deleting the gene encoding its inhibitory subunit Bcy1 did not increase Maf1 phosphorylation in *sch9Δ* (Figures 1A and 1B). Phosphorylation of Maf1 by PKA and Sch9 has been reported to regulate Maf1 nucleus-to-cytoplasm translocation and loss of Sch9 enriches Maf1 protein in the nucleus [27, 37]. We found that although *SCH9* deletion significantly enriched Maf1 in the nucleus, PKA hyper-activation did not reverse the nuclear localization (Figures 1C and 1D). Together, these results suggest that Sch9 but not PKA is the major kinase that phosphorylates and controls Maf1 subcellular localization *in vivo*. These observations further suggest that PKA and Sch9 are not redundant as thought before in Maf1 regulation.

### Hyper-activation of PKA decreases Maf1 protein abundance under growth inhibiting conditions

**Figure 2:**
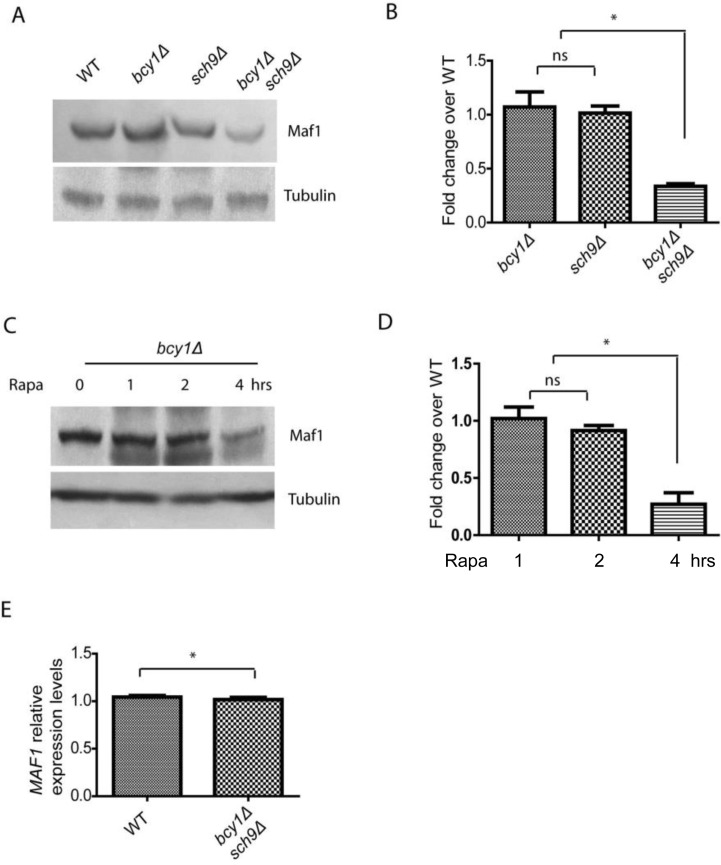
PKA and Sch9 coordinate to control Maf1 protein levels (**A**) Maf1 protein levels are reduced by PKA hyper-activation in sch9*Δ* cells. WT, *bcy1Δ*, *sch9Δ* and *sch9Δ bcy1Δ* cells expressing a Myc-tagged Maf1 (Maf1-Myc) from a low copy centromeric plasmid were cultured to early log phase and protein samples were prepared for western blot analysis. Protein amount were controlled by endogenous tubulin levels. (**B**) Quantification of Maf1 protein levels from 2 independent experiments as shown in A. ns, not significant; * *p* < 0.01. (**C**) Maf1 protein levels are reduced by PKA hyper-activation in growth inhibiting conditions. *bcy1Δ* cells expressing a Myc-tagged Maf1 (Maf1-Myc) from a low copy centromeric plasmid were treated with 100 nM rapamycin for 1, 2, 4 hours and protein samples were prepared for western blot analysis. Protein amount were controlled by endogenous tubulin levels. (**D**) Quantification of Maf1 protein levels from 2 independent experiments as shown in C. ns, not significant; * *p* < 0.01. (**E**) MAF1 mRNA levels are not reduced by *sch9Δ bcy1Δ* as shown by real-time qPCR.

Although Maf1 phosphorylation and subcellular distribution were not changed by PKA hyper-activation in *sch9Δ* cells, we noticed that Maf1 protein levels could be reduced (Figure 1A). To confirm this, we did the experiment without adding phosphatase inhibitors and incubated the sample with Calf Intestine Phosphotase (CIP), allowing accurate comparison of protein amount (Figure 2). The results demonstrated that Maf1 protein is indeed decreased in *sch9Δ bcy1Δ* double mutant, but neither single mutants (Figures 2A and 2B), suggesting a coordinated control of Maf1 by Sch9 and PKA. Since Sch9 is an effector of the central growth controller TORC1 [3], it is possible that in conditions where growth is inhibited, Maf1 is subjected to PKA regulation through protein levels. To test this, we treated yeast cells with rapamycin, a pharmacological compound that targets TOR therefore halts growth, and examined the Maf1 protein over time. Consistently, we found that Maf1 protein was gradually reduced in *bcy1Δ* mutant after rapamycin treatment (Figures 2C and 2D).

We further asked if the reduction in Maf1 protein abundance reflected mRNA levels or was regulated at the posttranslational levels. We did quantitative RT-PCR and found that MAF1 mRNA levels were not changed in *sch9Δ bcy1Δ* mutant compared to WT strain (Figure 2E). Therefore, PKA and Sch9 coordinate to regulate Maf1protein abundance at the posttranslational levels. The detail mechanism remains currently unknown.

### Constitutive PKA activity or loss of Maf1 prevents stress resistance and lifespan of *sch9* mutant

**Figure 3:**
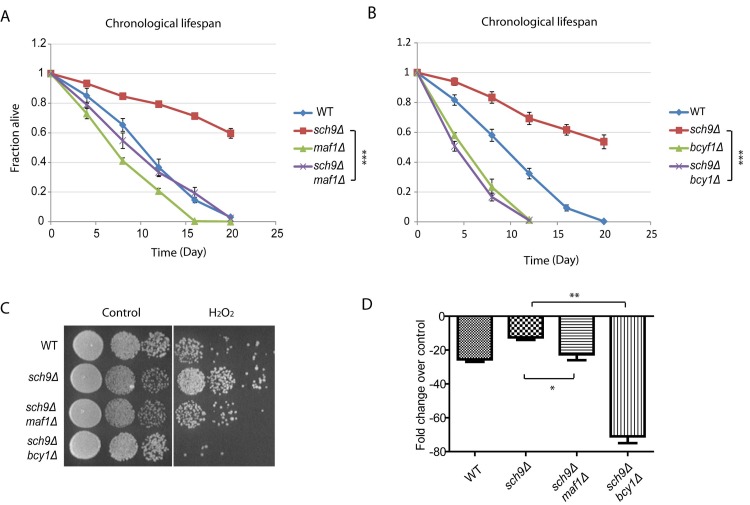
Maf1 mediates lifespan regulation and stress resistance of sch9*Δ* mutant (**A**) The extended chronological lifespan of sch9*Δ* mutant is shortened by *MAF1* deletion. WT, *sch9Δ* and *sch9Δ maf1Δ* cells were cultured to stationary phase and the fractions of survival over indicated time points were measured by colony forming assay. Error bars stands for the SEM of 3 replicates. *** *p* < 0.0001. (**B**) *BCY1* is required for sch9*Δ* to extend chronological lifespan. WT, *sch9Δ* and *sch9Δ bcy1Δ* cells were cultured to stationary phase and the fractions of survival over indicated time points were measured by colony forming assay. Error bars stands for the SEM of 3 replicates. *** *p* < 0.0001. (**C**) MAF1 and BCY1 are required for stress resistance of sch9Δ mutant. WT, sch9Δ, *sch9Δbcy1Δ* and *sch9Δmaf1Δ* cells were cultured to stationary phase and treated without or with H_2_O_2_ for 30 min. Cells were serial diluted and spotted on YPD plates and incubated for 2–3 days at 30°C. (**D**) Quantification of H_2_O_2_ resistance from 3 independent experiments as shown in C. Error bars stands for the SEM. ns, not significant; * *p* < 0.01, ** *p* < 0.01.

*sch9Δ* cells has extended chronological lifespan [8], since Maf1 is subject to Sch9 regulation, we wondered if Maf1 could mediate signaling pathways to confer this phenotype. To test this, we created *sch9Δ maf1Δ* double deletion cells and compared its chronological lifespan (CLS) with those of wild-type (WT), *sch9Δ* alone or *maf1Δ* alone. Chronological lifespan is defined by the time of cells surviving in stationary phase. By using colony assay, we calculated the percentage of living cell over 3 weeks. Our results showed that *MAF1* deletion significantly decreased the CLS of *sch9Δ*, but only slightly decreased that of WT (Figure 3A). Since in *sch9Δ*, PKA hyper-activation reduced Maf1 protein levels (Figure 2), we wanted to know if PKA hyper-activation could also mitigate the extended lifespan of *sch9Δ*. The results demonstrated that PKA hyper-activation by *BCY1* deletion abrogated the CLS of *sch9Δ* (Figure 3B). It is interesting that *BCY1* deletion shortened the lifespan of *sch9Δ* to a greater extent than *MAF1* deletion (compare Figures 3A and 3B). This suggests that other unknown factors downstream of PKA are contributing to the extended lifespan of *sch9Δ*. Lifespan and stress resistance are highly correlative. We therefore also tested the requirement of Maf1 for stress resistance of *sch9Δ*. *sch9Δ* has been shown to highly resistant to H_2_O_2_ [38]. When *BCY1* was further deleted in *sch9Δ,* cells became highly sensitive to H_2_O_2_, consistent with *bcy1Δ* effect on abrogating the extended lifespan of *sch9Δ* (Figures 3C and 3D). In addition, loss of *MAF1* reduced the resistance to H_2_O_2_, suggesting that Maf1 contributes to the stress resistance of *sch9Δ*.

### Overexpression of Maf1 extends lifespan of *bcy1Δ*

**Figure 4:**
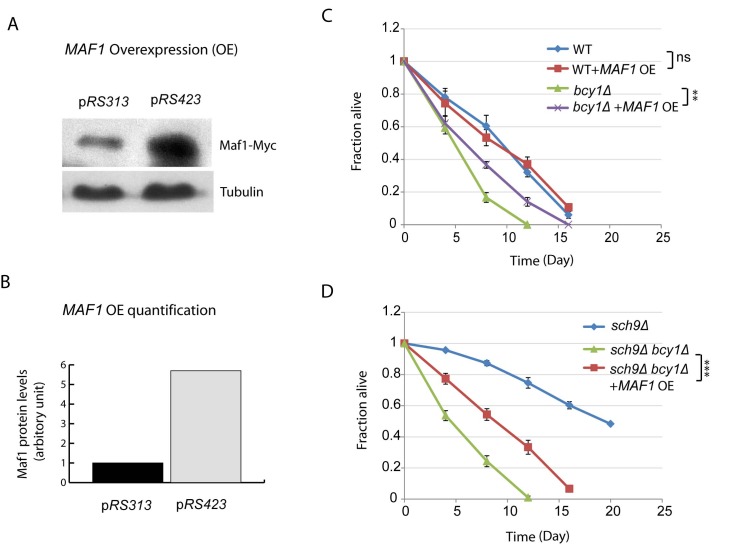
*MAF1* overexpression extends the short lifespan due to PKA hyperactivation (**A**) *MAF1* overexpression by using a high copy number plasmid increases the protein amount. *sch9Δbcy1Δ* cells expressing Myc-tagged Maf1 from low copy (p*RS313*) or high copy centrimeric plasmid (p*RS423*) were cultured to stationary phase and protein samples were prepared for western blot analysis. (**B**) Quantification shows around 6-fold increase in Maf1 protein expression in p*RS423*. (**C**) *MAF1* overexpression extends chronological lifespan of short-lived *bcy1Δ* mutant but not WT. WT and *bcy1Δ* cells without or with *MAF1* overexpression were cultured to stationary phase and the fractions of survival over indicated time points were measured by colony forming assay. Error bars stands for the SEM of 3 replicates. ns, not significant, **p < 0.001. (**D**) *MAF1* overexpression partially rescues the extended lifespan of *sch9Δ* mutant in *sch9Δbcy1Δ* cells. *sch9Δ* cells and *sch9Δbcy1Δ* cells without and with *MAF1* overexpression were cultured to stationary phase and the fractions of survival over indicated time points were measured by colony forming assay. Error bars stands for the SEM of 3 replicates, ***p < 0.0001.

Considering the importance of Maf1 in mediating Sch9 regulation of lifespan, we wanted to know if *MAF1* overexpression would be sufficient to extend lifespan. Although Maf1 is regulated at posttranslational levels, we were still able to sustain about 6-fold increase by using high copy number plasmid p*RS423* (Figures 4A and 4B). With such overexpression strategy, we did not observed any difference in lifespan for WT cells (Figure 4C). We reasoned that in the current assay for chronological lifespan, where nutrient has been largely depleted starting from day 3, Maf1 has already been activated; further increasing Maf1 protein amount may not further extend lifespan. To evaluate the importance of Maf1 abundance in CLS, we decided to use a mutant strain that is less sensitive to nutrient limitation. Hyper-activation of PKA by *BCY1* deletion can attenuate rapamycin to down-regulate ribosome biogenesis and growth [39], demonstrating that this strain can bypass the controls by TORC1 and become less sensitive to nutrients. We therefore overexpressed *MAF1* in *bcy1Δ* cells. The results showed that Maf1 significantly extended the chronological lifespan (Figure 4C). Since Maf1 protein levels were decreased in *sch9Δ bcy1Δ*, we also evaluated whether overexpression of Maf1 in this mutant strain could rescue the extended lifespan of *sch9Δ*. The results showed that lifespan of *sch9Δ bcy1Δ* can be extended, albeit not as long as that of *sch9Δ* (Figure 4D). The partial rescue of *sch9Δ* lifespan by overexpressing Maf1 in *sch9Δ bcy1Δ* confirms that Maf1 is an important lifespan regulator. It further suggests that PKA coordinate with Sch9 to regulate lifespan through factors in addition to Maf1.

### Constitutive PKA activity rescued Pol III activity and growth in *sch9Δ*, but is not due to Maf1 protein abundance

**Figure 5:**
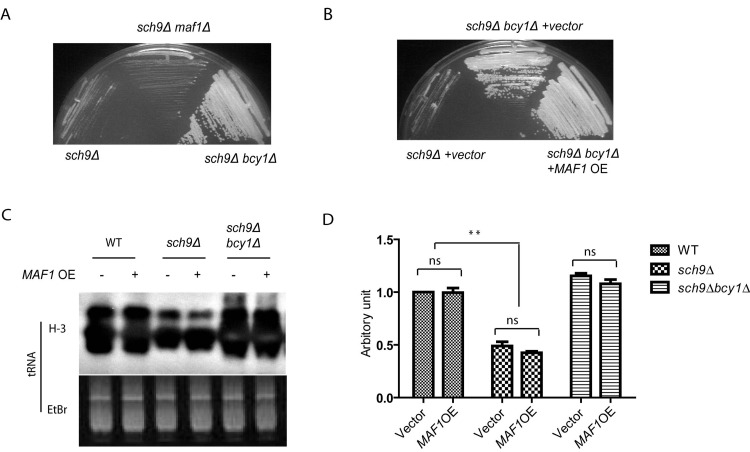
Maf1 is not involved in growth control by PKA and Sch9 (**A**) The slow growth phenotype of *sch9Δ* cells is reversed by PKA hyperactivation (*bcy1Δ*) but not *MAF1* deletion (*maf1Δ*). Yeast cells were streaked on YPD plates and incubated at 30 °C for 2 days. (**B**) The fast growing phenotype of *bcy1Δ* cells is not suppressed by overexpressing *MAF1 (MAF1* OE). Yeast cells were streaked on SD plate with Histidine dropout and incubated at 30 °C for 3 days. (**C**) MAF1 overexpression does not affect tRNA synthesis in WT, sch9Δ and sch9Δbcy1Δ cells. Yeast cell growing to early log phase were metabolic labeled with 3H. Total RNA was extracted and separated by poly acrylamide gel and stained with Ethidium bromide (Etbr) as loading control. Gel was dried and autoradiographed for newly synthesized tRNA (H-3). (**D**) Quantification tRNA synthesis of two separate experiments shown in C. ns, not significant, ** *p* < 0.001.

Though PKA and Sch9 are not redundant for Maf1 regulation, the slow growth phenotype associated with loss of Sch9 can be suppressed by enhanced PKA activity (Toda*et al*., 1988). We asked if Maf1 could also contribute to such growth suppression since Maf1 is decreased in protein levels in *sch9Δbcy1Δ*. If so, loss of Maf1 should mimic PKA hyper-activation and suppresses the slow growth of *sch9Δ* cells. Interestingly, although *MAF1* deletion partially suppressed stress resistance of *sch9Δ* cells (Figures 3C and 3D), loss of Maf1 did not suppress the slow growth of *sch9Δ* (Figure 5A). We also tested if the suppression of slow growth of *sch9Δ* by PKA could be reversed by *MAF1* overexpression. Again, although Maf1 overexpression partially rescued the extended lifespan *sch9Δ* in the short-lived *sch9Δbcy1Δ* (Figure 4D), such modulation did not rescue the slow growth phenotype (Figure 5B). This is also consistent with the observations that although PKA hyper-activation blocked *SCH9* deletion to reduce Polymerase III-dependent transcription of tRNA genes, overexpressing *MAF1* in such condition did not reverse the blockage (Figures 5C and 5D). Altogether, we conclude that Maf1 protein stability is not implicated in regulation of growth. Therefore, Maf1 acts to mediate PKA and Sch9 signaling only for stress resistance and lifespan modulation. These observations show that growth control and lifespan regulation can be uncoupled.

## DISCUSSION

### PKA and Sch9 do not function redundantly in Maf1 regulation

Since both PKA and Sch9 can phosphorylate Maf1 at similar amino acids *in vitro* [35, 37], an interesting question had been whether PKA was functionally redundant to Sch9. It was reported more than two decades ago that PKA hyper-activation can compensate for the loss of Sch9 for growth [40]. Later, microarray experiment demonstrated that these two kinases control essentially distinct sets of genes despite some overlaps [41]. Therefore, it is likely PKA functions redundantly to Sch9 only for certain but not all biological processes. For example, autophagy can only be induced by simultaneously inactivating both kinases [42], suggesting a possible redundancy in autophagy regulation. As to Maf1 regulation, however, although loss of Sch9 reduces Maf1 phosphorylation significantly [35–37], loss of all PKA catalytic subunits (lethal but rescued by *MSN2* and *MSN4* deletion) does not affect its phosphorylation at all [27]. In addition, although hyper-activation of Sch9 through mutating 8 Serine amino acids to Glutamate/Glutamine (2D3E mutant) prevents rapamycin to reduce Maf1 phosphorylation [37], hyper-activation of PKA by depleting Bcy1, the PKA regulatory subunit, does not [27, 35]. Therefore, likely the prevailing idea that PKA and Sch9 are both important for Maf1 phosphorylation could be misled by the *in vitro* assay. Care should be taken to interpret the *in vitro* kinas assay, as many kinases are promiscuous, but become specific *in vivo* due to restricted subcellular localization or/and defined regulatory subunits. To finally address this issue, we constitutively activate PKA in *sch9Δ* mutant by deleing *BCY1* and ask if PKA can compensate for the loss of Sch9 for Maf1 regulation *in vivo*. The results demonstrate that this is not the case, as neither the reduced phosphorylation nor the nuclear localization of Maf1 in *sch9Δ* is reversed by hyper-activated PKA. Together with evidence from other groups, our data demonstrate that PKA and Sch9 do not function redundantly as previously thought in Maf1 regulation.

### PKA and Sch9 cooperate to modulate Maf1 protein levels

Despite the fact that PKA and Sch9 are distinct in Maf1 regulation and probably many other biological processes, they have common targets [41]. For example, PKA and Sch9 has been known to cooperatively regulate autophagy: autophagy is induced in cells when PKA and Sch9 are simultaneously inactivated [42]. It is also known that both PKA and Sch9 are essential regulators of Polymerase III-dependent transcription [27, 37]. Since Maf1 is an inhibitor of RNA polymerase III activity in response to various growth and stress signals, it is not surprising that Maf1 had been proposed to be regulated redundantly by these two kinases. Although we found PKA and Sch9 do not regulate Maf1 through the same mechanism, we here discovered that in response to Sch9 loss or rapamycin treatment, which also inhibit Sch9, PKA hyper-activation decreases Maf1 protein amount. Therefore, instead of acting through the same mechanisms (phosphorylation hence subcellular distribution) to modulate Maf1 activity as that of Sch9, PKA may compensate for the loss of Sch9 through a new mechanism, that is, Maf1 protein stability. To our knowledge, this is the first report of Maf1 regulation through protein stability. Maf1 protein abundance is likely contributed by posttranslational regulation as MAF1 mRNA is not affected in this specific situation. The detail mechanism underlying Maf1 protein stability remains unknown. However, direct regulation of Maf1 by PKA seems unlikely because mutating all the putative PKA target sites on Maf1 does not affect Maf1 degradation (Data not shown). We propose that PKA indirectly modulates Maf1 stability by activating protein quality control machinery such as proteasome or specific protease. Also, whether Maf1 protein quality control is implemented in biological processes in addition to stress resistance and lifespan regulation (discussed hereafter), such as cell cycle progression, is intriguing but awaits further investigation.

### PKA modulates Maf1 protein abundance for stress and lifespan but not growth

The finding that PKA decreases Maf1 protein stability in growth inhibiting condition could have suggested a model to explain the compensation for Sch9 depletion by PKA hyper-activation: loss of Sch9 activates Maf1 through phosphorylation and nuclear enrichment while hyper-activation of PKA antagonizes such activation by down-regulating Maf1 protein amount. However, this model is only partially supported by our data. On the one hand, this model is plausible in that in *sch9Δbcy1Δ* double mutant where Maf1 protein is reduced, overexpression of Maf1 rescues the stress resistance and lifespan of *sch9Δ* mutant, albeit partially. On the other hand, this model is not impeccable with respect to growth, because rescued expression of Maf1 in *sch9Δbcy1Δ* double mutant does not rescue the reduced tRNA synthesis and the slow growth phenotypes of *sch9Δ* mutant. Growth and longevity are two major traits of biology and it has been noticed over decades to be inversely correlated [43]. Part of the theory underlying such correlation, termed “disposable soma theory of aging” [44], proposes that energy that is not expended for growth or reproduction will be allocated for soma maintenance, hence stress resistance and longevity. Although this holds true in the scope of natural evolution, our data suggest that under certain genetic or environmental conditions, these two major biological traits can be decoupled. The dissociation of growth/reproduction from stress response/lifespan is not without precedents, as while removing the germ cells in many organisms can extend lifespan, removing the whole reproduction system does not [45]. Our data have therefore provided another line of critical evidence encouraging new perspectives on this long-held aging theory.

## MATERIALS AND METHODS

### Strains and plasmids

Yeast cells were grown in either standard YPD (2% glucose, 2% peptone, 1% yeast extract) or synthetic defined (SD) medium with appropriate amino acid dropouts. DBY746 (MATa *leu 2-3,112 his3Δ1 trp1-289 ura3-52 GAL+*) and sch9D (MATa *leu 2-3,112 his3Δ1 trp1-289 ura3-52 GAL+ sch9::URA*) strains are originally from Dr. Longo lab at University of Southern California. All genetic manipulations are based on these two strains. *bcy1Δ* (MATa *leu 2-3,112 his3Δ1 trp1-289 uraz3-52 GAL+ bcy1::TRP*); *sch9 Δ bcy1Δ* (MATa *leu 2-3,112 his3Δ1 trp1-289 ura3-52 GAL+ sch9::URA bcy1::TRP*); *maf1Δ* (MATa *leu 2-3,112 his3Δ1 trp1-289 ura3-52 GAL+ maf1::KMAX4*); *sch9Δ maf1Δ* (MATa *leu 2-3,112 his3Δ1 trp1-289 ura3-52 GAL+ sch9::URA maf1::KMAX4*). To detect Maf1 protein at endogenous levels, *MAF1-MYC9* with its native promoter was inserted into low copy centromeric plasmid pRS313. To obtain high levels of Maf1 protein, *MAF1-MYC9* with its native promoter was inserted into high copy centromeric plasmid pRS423.

### Western blot

Yeast cells were collected by centrifugation then broken by glass beads by vigorous beating at 4°C in lysis buffer (50mM Tris-HCl pH7.5, 150mM NaCl, 0.5 mM EDTA, 0.5% NP-40, 2mM PMSF, Roche protease Complete inhibitor cocktail and phosSTOP tablet). Crude lysates were cleared by centrifugation and the supernatants were boiled in loading buffer. Protein samples were subjected to SDS-PAGE and transferred to membrane. For Western blotting, membrane was blocked by 1X TBST with 5% of non-fat milk, followed by incubating with mouse monoclonal anti-myc (9E10) antibody at the dilution of 1:5000 for at least 1 hour. Membrane was washed 3 times, 5 min each time by 1X TBST to remove non-specific binding. Membrane was then incubated with horseradish peroxidase (HRP)-conjugated secondary antibody for 30 min in 1X TBST with 5% of non-fat milk. Membrane was washed again by 1X TBST 3 times, 5 min each time before detection by enhanced chemiluminescence (ECL).

### Immunofluorescence

Yeast cells cultured to early log phase (OD_600_ = 0.2) were fixed by 3.7% formaldehyde for 1 hour. Cells were collected and washed with phosphate buffer (0.5 mM MgCl2, 40mM KH2PO4-K2HPO4 pH6.5), re-suspended in potassium buffer containing 1.2M sorbitol, and treated with 50 μg/mL zymolyase to obtain spheroplasts. Spheroplasts were immobilized on glass slide with cold methanol followed by acetone. Glass slides were dried and blotted with 1X PBS buffer containing 5% BSA then incubated with primary antibody (9E10, 1:100) for 1 hour. Surplus primary antibody was washed away by with 0.5% of Triton X-100 for 3 times. Glass slides were then incubated with secondary antibody for 30 min followed by wash with 0.5% of Triton X-100 for 3 times. Yeast cells were covered with cover slides and sealed with cytoseal 60 before imaging.

### Lifespan assay

Overnight yeast cultures (3 replicates for each sample) were diluted to OD_600_=0.2 and allowed to growth for 3 days to reach stationary phase, which was set to be day 0 for lifespan assay. Cultures were removed from flask every 4 days starting from day 0 and live cells were quantified by colony forming assay. For colony forming assay, removed cultures were 10X serial diluted and spread on YPD plates; colonies were allowed to form then quantified to obtain the average. Fractions of survival compared to that of day 0 (100% survival) were calculated and plotted.

### Real time quantitative PCR

Yeast cells were cultured to early log phase (OD_600_ = 0.4) and collected by centrifuge. Total mRNA was extracted by hot phenol method [46]. RNA was reverse-transcribed using QIAGEN One-Step RT-PCR Kit. Quantitative real time PCR was performed using the DNA Engine Opticon 2 (BioRad). Gene expression levels were normalized to actin (*ACT1*) and expressed as the percentage of wild type. Primers for *MAF1*: Forward, ttacaatgctacccttcagcaa; Rreverse, gagcagggtgattggtttgt. Primers for *ACT1*: forward, agctccaatgaaccctaaatca; reverse, acgacgtgagtaacaccatcac.

### Metabolic labeling

Metabolic labeling has been shown before [47]. Briefly, yeast cells in SD-Leu plus one third of the normal uracil were grown to OD_600_ = 0.4, then collected and washed with SD-Ura, re-suspended in 0.5 mL SD-Ura plus 15μCi/mL [5, 6-^3^H]-Uracil and incubated at 30 ℃ for 5 min. Total RNA was extracted by hot phenol method [46] and separated on 10% poly acrylamide gel containing 6M Urea, and analyzed by Ethidium bromide staining and ^3^H autoradiography.

### Statistical analysis

ImageJ was used to quantify signals for western blotting results and metabolic labeling. Prism 5.0 (Graphpad) was used for statistical analysis. *P* values were derived from student's *t*-test, two tailed.
